# Leaf‐Inspired Patterned Organohydrogel Surface for Ultrawide Time‐Range Open Biosensing

**DOI:** 10.1002/advs.202207702

**Published:** 2023-02-12

**Authors:** Hongxiao Gao, Xizi Wan, Yuemeng Yang, Jingwei Lu, Qinglin Zhu, Li‐Ping Xu, Shutao Wang

**Affiliations:** ^1^ Beijing Key Laboratory for Bioengineering and Sensing Technology School of Chemistry and Biological Engineering University of Science and Technology Beijing Beijing 100083 P. R. China; ^2^ CAS Key Laboratory of Bio‐inspired Materials and Interfacial Science Technical Institute of Physics and Chemistry Chinese Academy of Sciences Beijing 100190 P. R. China; ^3^ University of Chinese Academy of Sciences Beijing 100049 P. R. China

**Keywords:** biomarker detections, patterned organohydrogel surfaces, ultrawide time ranges, water retention

## Abstract

Droplet arrays show great significance in biosensing and biodetection because of low sample consumption and easy operation. However, inevitable water evaporation in open environment severely limits their applications in time‐consuming reactions. Herein, inspired by the unique water retention features of leaves, it is demonstrated that an open droplet array on patterned organohydrogel surface with water evaporating replenishment (POWER) for ultrawide time‐range biosensing, which integrated hydrophilic hydrogel domains and hydrophobic organogel background. The hydrogel domains on the surface can supply water to the pinned droplets through capillary channels formed in the nether organohydrogel bulk. The organogel background can inhibit water evaporation like the wax coating of leaves. Such a unique bioinspired design enables ultrawide time‐range biosensing in open environment from a few minutes to more than five hours involving a variety of analytes such as ions, small molecules, and macromolecules. The POWER provides a feasible and open biosensing platform for ultrawide time‐range reactions.

## Graphical abstract

The authors prepare a novel droplet array on patterned organohydrogel surface with water evaporating replenishment (POWER), enabling ultrawide biosensing time from minutes to hours with negligible sample diffusion. The POWER platform can be applied to almost all biosensing with different time ranges in an open environment, shedding new light on the design of next‐generation water retention platforms without additional equipment appendix.

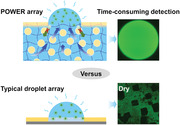

## 1 Introduction

Droplet arrays have attracted intensive attention in the fields of cell culture,^[^ ^1^ ^]^ individual microreactors,^[^ ^2^ ^]^ and biodetection^[^ ^3^ ^]^ by stockpiling specific nutrients, reactive molecules, or biomarkers. Their open structure and spatially ordered arrangement make them excellent miniaturized platforms for high‐throughput screening^[^ ^4^, ^5^ ^]^ and detection of (bio‐)chemical molecules. For droplet array‐based biosensing, both fast evaporation/enrichment^[^ ^6^ ^]^ and long‐term, continuous biodetection are important and have their own market demand. For those time‐consuming (bio‐)chemical reactions, multiple sequential steps are often involved and undergo long time spans,^[^ ^7^, ^8^ ^]^ placing high demands on the perdurability of droplet arrays. However, limited by inevitable water evaporation in the open environment, most droplet arrays are only applicable for short‐time reactions.^[^ ^9^, ^10^, ^11^, ^12^ ^]^ For those time‐consuming reactions, insufficient reactions and forced termination of the detection process often occurred due to water loss. This is because conventional approaches to constructing droplet arrays are often based on inorganic materials such as glass,^[^ ^13^ ^]^ silicon dioxide,^[^ ^14^ ^]^ or patterned polymer films,^[^ ^15^, ^16^ ^]^ which fail to store and supply water to the pinned droplets. Recently, many efforts have been attempted to solve the problems of water evaporation for droplet arrays. For example, nonmiscible liquids such as mineral oil,^[^ ^17^, ^18^ ^]^ alkane^[^ ^19^ ^]^ and fluorocarbon oil^[^ ^20^ ^]^ were used to cover the droplet arrays to replace the original gas‐liquid interface, thus inhibiting water evaporation. An alternative strategy is to seal the droplet arrays with a glass coverslip or PDMS.^[^ ^21^ ^]^ However, most of these efforts are devoted to creating enclosed space and inhibiting evaporation, which might cause inevitable contamination or need sophisticated engineering processes and large‐scale devices. It remains a great challenge to explore a simple and feasible strategy to prepare droplet arrays with controllable water retention properties, thus meeting diverse requirements for ultrawide time‐range biosensing.

**Figure 1:**
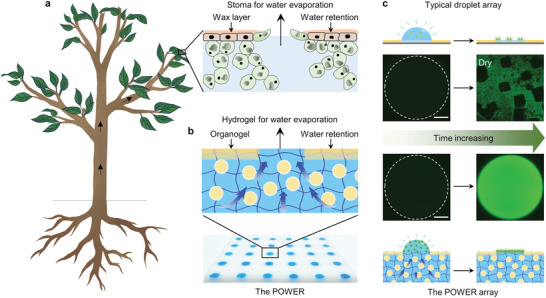
Bioinspired design of the POWER based on WET strategy. a) Schematic illustration showing the typical structure of leaves which combines wax layer for water retention and a stoma for water evaporation. b) Schematic illustration and optical image of a bioinspired POWER that integrates water‐retainable organogel domains and water‐passable hydrogel domains onto one surface. Moreover, capillary force in bulk organohydrogel facilitates water transport to prevent the POWER array from drying up. c) The POWER array can maintain for a long time, showing superiority over the typical superwettable droplet array in time‐consuming biosensing.

In nature, leaves exhibit unique on‐demand water retention functions.^[^ ^22^, ^23^ ^]^ The wax layer outside the epidermis, composed of hydrophobic compounds, is responsible for preventing water evaporation,^[^ ^24^ ^]^ and the stoma works as a gate to regulate water evaporation, thus adjusting to dynamic environmental conditions.^[^ ^25^ ^]^ Herein, inspired by the unique structure of leaves, we demonstrate a patterned organohydrogel surface with water evaporating replenishment (POWER) through a wetting‐enabled‐transfer (WET) strategy,^[^ ^26^ ^]^ which was composed of hydrogel and organogel domains on the surface and organohydrogel in the bulk (**Figure** **1**). Attributed to its unique structure, the obtained POWER presents integrated features including robust droplet‐pinning ability, desired water‐transport efficiency, and excellent water retention performance, realizing a much longer detection time of 5.6 h compared with ≈20 min for typical patterned superhydrophilic/superhydrophobic substrate at 20% humidity. Different from previously reported methods dependent on preventing water evaporation, our work proposes a replenishment approach to realize long‐time biodetection by providing an additional water supply. This bioinspired water retention strategy provides an alternative to developing next‐generation ultrawide time‐range biodetection platforms.

## 2 Results and Discussion

### 2.1 Bioinspired Design of the POWER

As an important component of most plants, leaves are ubiquitous in nature and have evolved ingenious stoma structure^[^ ^27^ ^]^ as well as wax layer for controllable moisture management (Figure 1a). Stoma distributed in the leaf epidermis can effectively regulate the water content during transpiration.^[^ ^28^, ^29^ ^]^ Wax layer composed of hydrophobic components is the main barrier against moisture loss for leaves.^[^ ^30^ ^]^ To mimic the unique structure of leaves for excellent water retention performance, hydrophobic organogel and hydrophilic hydrogel are selected and integrated into one surface through a WET strategy, resulting in the POWER. An oil‐in‐water emulsion containing organogel precursors in oil phase and hydrogel precursors in water phase was dripped and came into contact with the solid surface with hydrophobic/superhydrophilic pattern. Specifically, hydroxyethyl acrylate (HEA) with high hydrophilicity and neutral charge was chosen as the hydrogel monomer to reduce the impact on the detection of charged analytes. Lauryl methacrylate (LMA) was employed as the organogel monomer for its long‐alkyl chain to provide hydrophobicity and mechanical flexibility. After interfacial assembly through hydrophilic/hydrophobic interactions at the solid‐liquid interface and in‐situ emulsion polymerization, we can obtain a leaf‐like POWER of PHEA@PLMA organohydrogel integrating hydrophilic hydrogel domain with hydrophobic organogel domain on one surface. Hydrogel domain on the surface can function as a stoma for water entrapment and transport, organogel domain can act as the wax layer to inhibit water evaporation (Figure 1b). Meanwhile, the capillary channels of hydrogel in the nether organohydrogel bulk provide a driving force for efficient and continuous water supply.^[^ ^31^ ^]^ The surface and internal structure synergistically contribute to the excellent water retention property of the bioinspired POWER. As a proof of concept, Rhodamine 6G (R6G) droplets (in 1 × PBS buffer) were dripped on the bioinspired POWER surface to form the POWER array for fluorescence detection, taking typical superhydrophilic/superhydrophobic substrate as a contrast (Figure 1c). The time‐lapse fluorescence images showed that the POWER array presented a uniform and bright fluorescence image even after 120 min of evaporation (25 °C, 50% humidity). In sharp contrast, the droplet array on typical superhydrophilic/superhydrophobic substrate (typical superwettable droplet array) dried up and there appeared apparent crystals (rectangular black areas) on the surface after 30 ± 3 min, which was attributed to fast water evaporation in the open environment. Therefore, the as‐prepared bioinspired POWER with preferable water retention capacity provides a promising possibility for time‐consuming biodetection.

### 2.2 Structure and Wettability Characterization of the POWER

**Figure 2:**
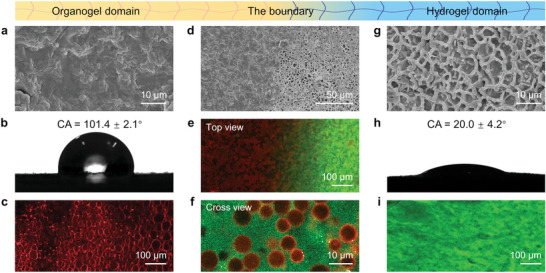
Structure and wettability characterization of the POWER. a) SEM image, b) water contact angle (WCA), and c) CLSM image of organogel domain on the POWER. Dense structure and hydrophobic properties contribute to the water retention performance of organogel domain. d) SEM image and e) CLSM image of the boundary domain. f) CLSM image showing the internal structure of the POWER, which was composed of continuous hydrogel phase and dispersed organogel particles. g) SEM image, h) WCA, and i) CLSM image of hydrogel domain on the POWER. Porous structure and hydrophilic properties are conducive to water‐pinning and transport performance of hydrogel domains.

The locally differentiated wettability and unique structure are critical for pinning droplet array and realizing water retention performance of the POWER. We, therefore, characterized the microstructure and surface wettability of the POWER through scanning electron microscope (SEM), confocal laser scanning microscope (CLSM) and static water contact angle (WCA) measurement. The induced organogel domain showed a smooth and nonporous structure (**Figure** **2**a) as well as hydrophobicity with a WCA of 101.4 ± 2.1° (Figure 2b). CLSM image also confirmed its hydrophobicity as the organogel domain can be dyed by hydrophobic fluorescent dye (1,1’ ‐dioctadecyl‐3,3,3’,3’‐tetra‐methylindocarbocyanine perchlorate, DiI, red) (Figure 2c). Both dense structure and hydrophobicity are conducive to the water retention property of the POWER (Figure S1, Supporting Information). In comparison, the induced hydrogel domain presented a microporous structure (Figure 2g) and hydrophilicity with a WCA of 20.0 ± 4.2° (Figure 2h). Hydrophilic fluorescent dye Rhodamine 110 could be absorbed in this region (Figure 2i). The microporous structure and hydrophilicity synergistically contribute to the water‐pinning and water transport capability of the POWER. From organogel to hydrogel domain, a clear boundary in structure and wettability can be observed from SEM and CLSM images (Figure 2d,e). X‐ray photoelectron spectroscopy revealed that the atomic percent of C and O were 81.7% and 14.7% for the organogel domain, and 69.5% and 21.5% for the hydrogel domain (Figure S2, Supporting Information), indicating their distinct difference in chemical composition. For the internal structure of the POWER, CLSM image showed a biphasic structure composed of a continuous hydrogel phase wrapping around organogel particles with a diameter of 5–20 µm (Figure 2f). Exploiting the unique structure, we constructed the POWER array and performed droplet stability test by rotating the POWER with angles of 0°, 90°, 135° and 180°. The droplet array on the hydrogel domain can be firmly pinned without falling at an arbitrary rotation angle, demonstrating the robustness of the POWER array (Figure S3a, Supporting Information). Additionally, we explored the influence of hydrogel‐domain diameter on the maximum volume of pinned droplets. The maximum volume increased from 5 µL to 30 µL with the diameter of hydrogel domain increasing from 1 mm to 4 mm (Figure S3b, Supporting Information). Therefore, the bioinspired POWER possesses surface‐patterned organogel and hydrogel domains and exhibits robust droplet‐pinning capacity.

### 2.3 The Water Retention Behavior of the POWER

**Figure 3:**
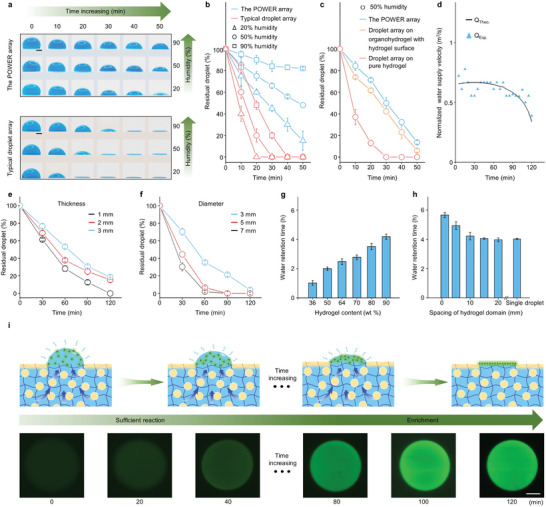
Water retention behavior of the POWER and its influence factors. a) Time‐lapse images showing the volume change of droplets on the POWER (3 mm in diameter, 3 mm in thickness), taking typical superhydrophilic/superhydrophobic substrate as a contrast. Scale bar, 1 mm. b) Comparison of residual droplets on the POWER and typical superhydrophilic/superhydrophobic substrate (3 mm in diameter, 3 mm in thickness) during a 50 min recording under humidity from 20%, 50%, and 90%. c) Comparison of water retention capability between the organohydrogel with hydrogel surface, pure hydrogel, and the POWER (5 mm in diameter, 3 mm in thickness). d) Comparison of normalized experimental water supply velocity (*Q*_Exp._, blue triangles) and theoretical water supply velocity (*Q*_Theo.,_ black line) as a function of time. e) Influence of the thickness (3 mm in diameter, 25 °C, 50% humidity). f) Hydrogel‐domain diameter (3 mm in thickness, 25 °C, 50% humidity) on the water retention property of the POWER. g) Water retention time of the POWER platform with different hydrogel contents from 36 wt.% to 90 wt.%. h) Water retention time of the POWER (90 wt.% hydrogels) with different hydrogel‐domain spacings. Decreased hydrogel‐domain spacing leads to less evaporation and longer water retention time. i) Schematic illustration of water retention process and corresponding time‐lapse fluorescence images of R6G droplet on the POWER during 120 min. The temperature is maintained at 25 °C and humidity is 50% unless otherwise specified. Scale bar, 200 µm. Data represent the mean ± s.d. (*N*  =  3).

To investigate the water retention behavior of the POWER, we monitored the dynamic evaporation process of the POWER array (10 µL, labeled blue), taking the typical superwettable droplet array as a control. A series of time‐lapse images showing the volume change of droplets were captured under different humidity ranging from 20%, 50% to 90%. As shown in **Figure** **3**a, the POWER array can keep for 50 min without drying up in an open environment with different humidity. In comparison, the volume of the typical superwettable droplet array gradually decreased and approached almost zero within 20 ± 3 min at 20% humidity, 30 ± 3 min at 50% humidity, and 38 ± 4 min at 90% humidity, respectively. The quantitative analysis of residual droplet further confirms that the POWER shows superior water retention performance than typical superwettable substrate at different stages (Figure 3b), which may be ascribed to additional water supply from the nether bulk organohydrogel. To explore the effect of organogel background and bulk organogel particles on the water retention performance of the POWER, we compared the water retention behavior of the POWER, organohydrogel with hydrogel surface and pure hydrogel (Figure S4a, Supporting Information). Residual droplets on the POWER and organohydrogel with hydrogel surface remained over 40% after 30 min of evaporation. In comparison, under the same environmental conditions and evaporation time, the droplet array on pure hydrogel completely dried up (Figure 3c and Figure S4b, Supporting Information). In addition, the POWER shows better water retention performance than organohydrogel with hydrogel surface due to the organogel background on the surface inhibiting water evaporation. Therefore, the superior water retention performance of the POWER can be attributed to the synergistic effect of the barrier of the surface organogel domain and the additional water supply from the nether bulk organohydrogel.

To further verify the underlying mechanism of the superior water retention property of the POWER, we employed a simplified capillary model to explain the additional water supply as shown in Figure S5, Supporting Information. The POWER is a typical porous media in which the velocity *Q* (the volume of water supply per unit time) of the fluid flow can be calculated by Darcy's law,^[^ ^32^ ^]^

(1) $$Q\;=\;-\frac{k}{\mu }\frac{A\;\Delta P}{L}\;$$

Here, *k* is the hydraulic permeability (a parameter related to the pore size),^[^ ^33^ ^]^ *A* is the cross‐sectional area of the hydrogel domain, *μ* is the dynamic viscosity of water, *L* is the thickness of the POWER, and Δ*P* is the capillary force induced by curved droplet surface that can be calculated through the Laplace formula,^[^ ^34^, ^35^ ^]^

(2) $$\Delta P\;=\;\frac{2\gamma \;\cos\theta }{R}\;$$

Here, *γ* is the surface tension of water, *R* is the radius of the hydrogel domain, and *θ* is the WCA. Combining Equation (1) and Equation (2), the velocity of theoretical water supply from the nether bulk organohydrogel can be expressed as:

(3) $$Q_{Theo.}=\;-\;\frac{k}{\mu }\frac{2\pi R\;\gamma \;\cos\theta }{L}$$

In the case of the POWER, the velocity of water supply is only related to the WCA. As shown in Figure 3d, the theoretical water supply velocity (*Q*_*Theo*._) is consistent with the experimental data (*Q*_*Exp*._), where *Q*_*Exp*._ can be obtained by measuring the difference in the mass‐loss rate of the POWER array and typical superwettable droplet array. Therefore, the superior water retention performance of the POWER array probably originates from a porous capillary model of water supply.

We next investigated the influence of organohydrogel thickness, hydrogel content, hydrogel‐domain diameter, and spacing on the water retention property of the POWER. As illustrated in Figure 3e‐h, the water retention efficiency of the POWER increased with the increase of organohydrogel thickness and hydrogel content, however, decreased with the increase of hydrogel‐domain diameter and spacing. Thicker organohydrogel and higher hydrogel content provide more available water to the pinned droplets, while larger hydrogel‐domain diameter and spacing increase the evaporation rate of droplet arrays (Figure S6, Supporting Information).^[^ ^36^, ^37^ ^]^ With a high hydrogel content of 90 wt.% and small hydrogel domain spacing of 1 mm, the POWER platform exhibits a long water retention time of 5.6 ± 0.2 h, which is far superior to conventional open detection chips based on oxide, inorganic materials, organic polymers or metals (Table S1, Supporting Information).^[^ ^3^, ^15^, ^38^, ^39^, ^40^, ^41^, ^42^ ^]^ Further increase in hydrogel content is not conducive to forming a stable oil‐in‐water emulsion and the subsequent WET process. Deformation can also affect the water retention efficiency of the POWER platform to some extent. Stretching to twice the original length reduces the water retention time of POWER to 60% of the original, while squeezing by applying a vertical force extends the water retention time of POWER to 1.26 times the original (Figure S7, Supporting Information). In addition, during the continuous water supply process, the collapse in bulk organohydrogel structure due to water loss also affects the water retention property of POWER. Nevertheless, the water loss and network collapse process is very slow, during which the POWER can be left for more than 5 h without drying out (Figure S8, Supporting Information). It should be noted that such a long water retention time cannot be achieved by typical superwettable chips. Even if covering a lid or creating an enclosed space for them, the water retention time of superwettable chips can only be extended from the original 0.5 h to 2 h, which is much shorter than that of the POWER (Figure S9, Supporting Information). As a proof of concept, we dripped a drop of R6G (10 µL, 10 nM) on the POWER surface and recorded the fluorescence intensity of R6G with time. The fluorescence intensity showed an unnoticeable increase in the initial 60 min and an apparent enrichment subsequently (Figure 3h and Figure S10, Supporting Information). Such an ultrawide reaction time range and fast enrichment process are just crucial to long‐time biosensing.

### 2.4 Ultratrace Biomarker Detection with the Bioinspired POWER

**Figure 4:**
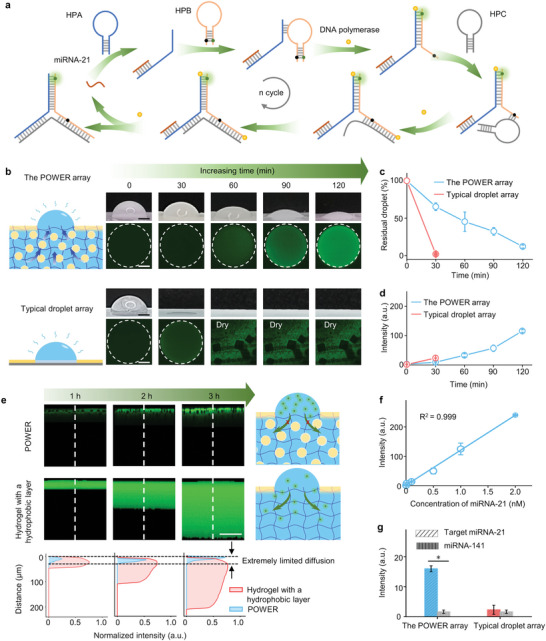
Ultratrace biomarker detection with the bioinspired POWER. a) Schematic illustration of the SDA reaction for miRNA‐21 detection. b) Schematic illustration of the POWER array compared with typical superwettable droplet array and corresponding time‐lapse fluorescence as well as optical images. Scale bars, 1 mm for optical images and 200 µm for fluorescence images. c) Quantitative analysis of residual droplet and d) fluorescence intensity during the reaction process. e) Schematic and cross‐view CLSM images showing the diffusion of rhodamine inside the POWER and hydrogel with a hydrophobic layer, respectively. Scale bar, 50 µm. f) Linear relationship between fluorescence intensity and the concentration of miRNA‐21 based on the POWER array with a detection limit of 0.4 pM. The regression equation was *I* = 2.014C + 118.958 with a correlation coefficient of 0.999. I is the signal intensity and C is the concentration of target miRNA‐21. g) Comparison of specificity of the POWER array and typical superwettable droplet array upon addition of target miRNA‐21 or miRNA‐141, respectively. *P* < 0.001 (*).

As an isothermal amplification strategy, the strand displacement amplification (SDA) reaction usually takes a long time to reach reaction plateau,^[^ ^43^, ^44^, ^45^ ^]^ placing high demands on the water retention capacity of traditional open biodetection platforms. As a proof of concept, we chose a typical SDA reaction for the detection of miRNA‐21 (120 min needed in the tube, Figure S11 and Table S2, Supporting Information) (**Figure** **4**). In brief, a 10 µL droplet containing 1 nM of miRNA‐21, 20 nM of hairpin probe A (HPA), hairpin probe B (HPB) and hairpin probe C (HPC) was dripped on the POWER, taking typical superwettable droplet array as a control. With the presence of target miRNA‐21, the fluorescence signal can be generated due to the formation of Y‐shaped DNA by HPA opening the hairpin structure of HPB. With time increasing, more Y‐shaped DNA formed, and target miRNA‐21 was released to initiate the next round of the reaction cycle, resulting in a gradual increase of fluorescent signals (Figure 4a). Time‐lapse optical and fluorescence images were recorded to characterize the volume and fluorescence intensity change over time (Figure 4b). The volume of the POWER array gradually decreased, but the droplet did not dry up within 120 min, which allowed sufficient SDA reaction. The latter enrichment further amplified the fluorescence signal on the POWER. In contrast, low fluorescence signal can be detected for the typical superwettable droplet array at the same target concentration. The rapid water evaporation leads to the early termination of SDA reaction and results in low amplification efficiency. Meanwhile, apparent salt crystals (rectangular black areas in CLSM images) can be observed after 30 min of evaporation. The corresponding volume and fluorescence intensity of residual droplets on the POWER and typical superhydrophilic/superhydrophobic substrate were shown in Figure 4c,d. Most importantly, the introduction of organogel particles to the bulk phase of POWER can effectively reduce the diffusion pathways of analytes,^[^ ^46^ ^]^ enabling a limited diffusion depth of less than 30 µm even after 3 h of standing, which is similar to that on typical superwettable chips composed of inorganic SiO_2_ (Figure S12, Supporting Information). In comparison, for a simple combination of hydrogel and a hydrophobic protective layer, a large molecular diffusion was observed up to 200 µm inside the pure hydrogel under identical conditions, which severely diluted the concentration of analytes and resulted in inaccurate biodetection (Figure 4e). Therefore, we believe that the POWER platform is irreplaceable by traditional hydrogel‐based detection chips, whether in terms of sufficient detection time or low molecular diffusion.

Utilizing the excellent properties of the POWER, we can realize the detection of ultratrace biomarkers without any additional equipment. According to the regression equation of miRNA‐21 detection from 1 pM to 2 nM, the limit of detection (LOD) was calculated to be 0.4 pM for the POWER (Figure 4f). Although this LOD is similar to the lowest value of the reported similar system for the detection of miRNA‐21 in tubes, the sample consumption greatly decreased from 200 µL to 10 µL (Table S3, Supporting Information). The specificity of miRNA‐21 sensor on the POWER was evaluated by introducing miRNA‐141 into the SDA reaction system. As miRNA‐141 cannot trigger HPA to open the hairpin structure of HPB and form fluorescent products, there appeared a significant difference in fluorescent intensity for the detection system of miRNA‐21 and miRNA‐141 after 120 min of reaction, showing high specificity. In contrast, a much lower fluorescence signal can be detected for the typical superwettable droplet array with either miRNA‐21 or miRNA‐141 as the target even in the concentration of 0.1 nM (Figure 4g). In addition, the POWER platform can also be used for high‐throughput detection without sample cross‐contamination due to the superior water retention ability and extremely low sample diffusion (Figure S13 and S14, Supporting Information). These results indicate that the POWER with preeminent water retention performance allows sufficient reaction time to realize accurate detection of ultratrace biomarkers.

### 2.5 Universality of the POWER in Biosensing

**Figure 5:**
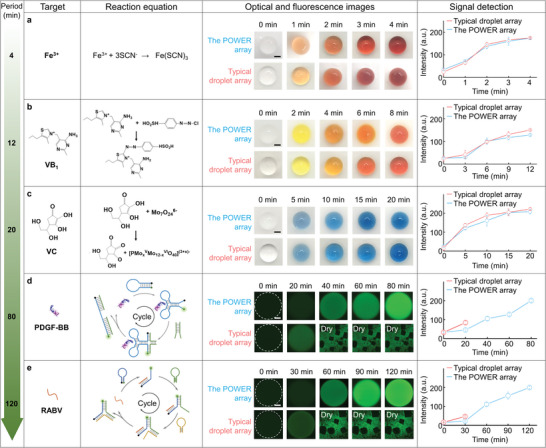
Comparison of analyte detection with different reaction times between the POWER array and typical superwettable droplet array. Detection of a) Fe^3+^ with a reaction time of 4 min, b) VB_1_ with a reaction time of 12 min, c) VC with a reaction time of 20 min, d) PDGF‐BB with a reaction time of 80 min and e) RABV with a reaction time of 120 min. Scale bars, 1 mm for a‐c and 200 µm for d, e. Data represent the mean ± s.d. (*N*  =  3).

To prove the universality of the POWER, we selected several typical biodetection systems undergoing different reaction times from 4 min to 120 min for a series of analytes from ions (Fe^3+^), small molecules such as Vitamin B_1_ (VB_1_), and Vitamin C (VC) to macromolecules (protein and nucleic acid). Optical images and fluorescence signals were recorded at different stages for these reaction systems and the typical superhydrophilic/superhydrophobic substrate was taken as a contrast. As shown in **Figure** **5**, the POWER array achieves reliable biomarker detection for all the analytes spanning an ultrawide range of time due to the sufficient water supply. In comparison, although the superwettable droplet array can fulfill the detection of short‐time reactions such as the Fe^3+^ (4 min), VB_1_ (12 min), and VC (20 min) detections (Figure 5a‐c), a detection interruption occurred after 30 min for those time‐consuming biological reactions such as the detection of platelet‐derived growth factor‐BB (PDGF‐BB) (80 min) and rabies virus (RABV) (120 min) due to dry‐up of water (Figure 5d,e). Notably, these molecules (R6G, DNA, and protein) can be easily washed off through ultrasound probably due to the weak bonding between the electroneutral HEA hydrogel and these charged molecules, demonstrating the durability and antifouling property of the POWER platform (Figure S15, Supporting Information). These results indicate that the POWER with excellent water retention ability is applicable to ultrawide time‐range biosensing, addressing the intractable detection failure due to liquid evaporation faced by typical open droplet arrays.

## 3 Conclusion

In summary, we demonstrated a droplet array on bioinspired POWER through a one‐step WET strategy, realizing ultrawide time‐range biomarker detection. The POWER shows several prominent advantages. First, a bioinspired water retention strategy can greatly extend the effective detection time of the POWER array by simultaneously inhibiting water evaporation on the surface and creating capillary channels for water supply in the bulk, behaving like the leaves. Second, the POWER offers an alternative replenishment mode to droplet arrays different from conventional enclosed‐space detection that heavily relies on complex operations or sophisticated equipment. Third, our bioinspired open droplet array enables the detection of most biological reactions involving ions, small molecules, and macromolecules, spanning ultrawide‐range time from minutes to hours with little sample diffusion. It can also be extended to a large variety of complicated and specific biodetection by selecting different gel precursors based on the interaction with different biological targets. Our open POWER system without any additional equipment appendix is irreplaceable by the traditional enclosed systems and has great prospects in some economically backward regions where healthcare is poor. This study showcases great potential for downstream applications associated with ultratrace biomarker detection, such as cancer diagnoses and infectious disease detection.

## 4 Experimental Section

### Preparation of the POWER

First, the glass substrates with patterned wettability were fabricated according to the following procedures: glass substrates were successively cleaned with acetone, ethanol and 1% NaOH (w/w) aqueous solution, and afterward, they were washed three times with DI‐water and dried with nitrogen. The hydrophobic glass substrates were fabricated via chemical vapor deposition of 1H,1H,2H,2H‐perfluorodecyltrimethylsilane in the vacuum environment at 80 °C for 6 h. Then the hydrophobic‐modified glass substrates were treated with O_2_ plasma through the photomask (circular pattern arrays with diameters of 3, 5, and 7 mm and spacing of 10 mm) for 5 min. The covered regions of glass surface remained hydrophobic, whereas the uncovered regions became superhydrophilic. Second, the POWER with 64 wt.% hydrogel content was synthesized by a WET strategy with an oil‐in‐water emulsion system. Hydrogel precursor ingredients including 120 g water, 17.1 g HEA, 30 mg 2,2‐diethoxy acetophenone (DEAP), and 4.8 g nanoclay were mixed and stirred for 1 h. Organogel precursor ingredients including 76.2 g LMA, 3 g ethylene glycol dimethacrylate (EGDMA) and 60 mg DEAP were mixed, and stirred as the oil phase. The two phases were mixed together and emulsified for 1 min to form stable emulsion. The oil‐in‐water emulsion composed of a two‐phase prepolymer solution was poured into the glass molds with patterned wettability. The organogel precursor tended to be adsorbed onto the hydrophobic regions of glass substrates through hydrophobic interaction, while hydrogel precursor was adsorbed onto hydrophilic regions of the glass substrates through hydrophilic interaction. Subsequently, in‐situ photopolymerization was performed under UV irradiation (20 mW cm^−2^ of light intensity) for 30 min. Organohydrogel with different thicknesses could be obtained by tuning the glass mold interval. Finally, the prepared POWER was peeled off from the glass substrates lightly.

### Statistical Analyses

The fluorescence and background intensities over each region were quantified using customized imaging software and ImageJ v1.52i (National Institutes of Health, Bethesda, MD). Presented data normalized in this manuscript are specified in the captions. Values and error bars represent the mean and standard deviations of measurements between replicates, respectively. Sample sizes are specified in the captions of the figures for each quantitated result. The differences among groups were determined using one‐way ANOVA analysis to assess the statistical differences. p values of less than 0.05 were considered to be statistically significant. The data was indicated with ***p < 0.05, **p < 0.01, and *p < 0.001.

## Conflict of Interest

The authors declare no conflict of interest.

## Supporting information

Supporting Information

## Data Availability

The data that support the findings of this study are available from the corresponding author upon reasonable request.
