# Supplementary material for: Leaf‐Inspired Patterned Organohydrogel Surface for Ultrawide Time‐Range Open Biosensing
Source: Adv Sci (Weinh). 2023 Feb 12;10(11):2207702. doi: 10.1002/advs.202207702 (PMC10104639; doi:10.1002/advs.202207702)
Supplement: Supplementary file 1 — Supporting Information [file ADVS-10-2207702-s001.pdf]

ADVANCED SCIENCE
Open Access

Supporting Information

for *Adv. Sci.*, DOI 10.1002/advs.202207702

Leaf-Inspired Patterned Organohydrogel Surface for Ultrawide Time-Range Open Biosensing

Hongxiao Gao, Xizi Wan, Yuemeng Yang, Jingwei Lu, Qinglin Zhu, Li-Ping Xu and Shutao Wang**

Supporting Information

Leaf-inspired patterned organohydrogel surface for ultrawide time-range open biosensing

Hongxiao Gao, Xizi Wan, Yuemeng Yang, Jingwei Lu, Qinglin Zhu, Li-Ping Xu* and Shutao Wang*

Table of contents

1. Chemicals.

2. Preparation of typical superhydrophilic/superhydrophobic substrates

3. Characterization of the POWER

   3.1 SEM characterization

   3.2 CLSM characterization

   3.3 XPS characterization

   3.4 CA measurements

4. Water retention behavior analysis of the POWER

5. Analyte detections

6. The molecule diffusion from the surface to the organohydrogel bulk

1. Chemicals

Inorganic nanoclay (Laponite XLS, $[Mg_{5.34}Li_{0.66}Si_8O_{20}(OH)_4]$ $Na_{0.66}$, layer size = 20-30 nm in diameter and 1 nm in thickness, MW = 762.24.) was purchased from Rockwood Ltd, UK, lauryl methacrylate (LMA), ethyleneglycol dimethacrylate (EGDMA), hydroxyethyl methacrylate (HEA), 2,2-diethoxyacetophenone (DEAP) and 1H,1H,2H,2H-perfluorodecyltrimethylsilane were bought from Aladdin Co., Ltd. (Shanghai, China).

Rhodamine 6G was bought from Aladdin Co. Ltd (Shanghai, China). Rhodamine 110 and 1,1'-dioctadecyl-3,3,3',3'-tetramethylindocarbocyanine perchlorate (DiI) were purchased from AmyJet Scientific Co., Ltd. (Wuhan, China). $FeCl_3$, KSCN, Vitamin B1 (VB1) and Vitamin C (VC) were purchased from Innochem Co., Ltd. (Beijing, China). Platelet-derived growth factor-BB (PDGF-BB) was purchased from Beijing T&L Biotechnology Co., Ltd. (Beijing, China). Klenow fragment (3’→5’ exo-) polymerase (KFP; 5 U μL^{-1}) and 1 × TBS buffer were bought from Sangon Biotech Co. Ltd (Shanghai, China). Deoxyribonucleoside 5’-triphosphates (dNTPs) were purchased from Yuanye Co. Ltd (Shanghai, China). All the chemical reagents were used directly without other purification. Oligonucleotides were synthesized by Sangon Biotech Co. Ltd (Shanghai, China). The environmental conditions were controlled by a Thermostatic-Humidistat cultivating box (Beijing Luxi Technology Co., Ltd., China).

2 Preparation of typical superhydrophilic/superhydrophobic substrates

Typical superhydrophilic/superhydrophobic substrates were synthesized using a similar approach according to reference.[1] In brief, the superhydrophilic silica nanocoating was constructed by the dip-coating method. Then the silica coating was immersed in a methylbenzene solution of octadecytrichlorosilane (OTS, 1wt.%) for 45 min to obtain superhydrophobicity. Superhydrophilic microwells were created by UV irradiation through a photomask-assisted method on the superhydrophobic surface. Thus typical superhydrophilic/superhydrophobic patterned substrates were prepared.

3 Characterization of the POWER

3.1 SEM characterization.

The POWER was immersed in liquid nitrogen and immediately treated with lyophilization at -85 °C for 3 days. Structure characterization was performed on a field-emission scanning electron microscope (Hitachi, SU8010). To enhance the electrical conductivity of samples, a thin gold particles film was coated on the POWER before observation.

3.2 CLSM characterization.

The hydrogel domains and organogel domains were stained by Rhodamine 110 and DiI (10 nM) respectively for 5 min. The excitation wavelengths were 492 nM and

549 nm respectively. The CLSM images were obtained from a laser scanning confocal microscope (Olympus, FV1000-IX81).

3.3 XPS characterization.

After treatment with lyophilization at -85 °C for 3 days, the XPS data of the POWER was obtained from a ThermoFisher Scientific ESCALAB 250Xi system.

3.4 CA measurements.

Water contact angles (WCAs) of the hydrogel domains and organogel domains on the POWER were tested by an OCA-25 machine (Dataphysics Germany) at ambient temperature. 3 uL of the droplets was used for the measurement of WCAs.

4 Water retention behavior analysis of the POWER

A typical analysis of water retention behavior for the POWER was described as follows: Firstly, the POWER was immersed in Milli-Q water to reach a swelling equilibrium state. The POWER array was prepared by dripping 10 μL of the blue-dyed droplet on the hydrogel domains. The POWER array was put on the water surface to ensure sufficient water supply. The residual droplets on the POWER denoted as C_W was calculated according to Equation S1:

$$C_W\% = \frac{\left(W_t - W_{g0} + \frac{(W_{cg0} - W_{cgt}) \times W_{g0}}{W_{cg0}}\right)}{W_{ow}} \times 100\% \quad \text{(S1)}$$

Where W_t is the real-time weight of the total platform including gel and the droplet, W_{g0} is the weight of the original gel, W_{cg0} and W_{cgt} are the original and real-time weights of control gel (the gel with the same parameters as the experiment gel for

quantifying the water loss of experimental gel) respectively, W_{ow} is the original weight of the droplet.

The residual typical superwettable droplet array was calculated according to Equation S2 :

$$C_W\% = \frac{(W_t - W_{s0})}{W_{ow}} \times 100\% \quad \text{(S2)}$$

Where W_t is the real-time weight of the total platform including typical superhydrophilic/superhydrophobic substrate and the droplet, W_{s0} is the weight of the typical superhydrophilic/superhydrophobic substrate. W_{ow} is the original weight of droplet.

5 Analytes detection

The POWER (3 mm in thickness and 3 mm in diameter) and typical superhydrophilic/superhydrophobic substrates (3 mm in diameter) anchoring 10 μL reaction droplets were prepared with the procedure described above and used for further detection. The prepared POWER was stored in DI water until use. The detection process was performed at 25 °C and 50% humidity unless otherwise specified. The fluorescence and optical images were obtained from a confocal laser scanning microscope and camera respectively. The intensity of fluorescence signals was determined with Image J software.[2]

For the detection of Fe^{3+}: 5 μL of $FeCl_3$ (24 μM) of and 5 μL of KSCN (120 mM) were mixed and dripped on the POWER and typical

superhydrophilic/superhydrophobic substrate.

For the detection of VB1: A reaction droplet (10 μL) containing 10 μM of VB1 and 3 mM of diazotized p-aminobenzene sulfonic acid in $NaHCO_3$ solution (0.1 M) was dripped on the POWER and typical superhydrophilic/superhydrophobic substrate.

For the detection of VC: A reaction droplet (10 μL) containing 10 μM of VC and 30 mM of ammonium molybdate solution was added to the sulfuric acid/phosphoric acid buffer (0.1 M, PH = 3.4) and dripped on the POWER and typical superhydrophilic/superhydrophobic substrate.

For the detection of miRNA-21: A reaction droplet containing different concentrations (0, 1 pM, 10 pM, 0.1 nM, 0.5 nM, 1 nM and 2 nM) of miRNA-21, 20 nM of HPA, HPB, HPC, 1 μL of dNTPs, 1 μL of KFP to a final volume of 10 μL in 10 × NEBuffer (PH = 7.9, 100 mM MgCl2, 100 mM Tris-HCl, 500 mM NaCl, 10 mM dithiothreitol) was dripped on the POWER and typical superhydrophilic/superhydrophobic substrate (Table S2, Supporting Information). The detection limit of miRNA-21 was calculated based on the blank signal plus 3 times the standard deviation of the blank signal.[3]

For the detection of PDGF-BB: A reaction droplet containing 50 nM of PDGF-BB, 200 nM of H1, DNA1, and DNA2 (Detailed sequences see Table S4, Supporting Information) in 10 μL of 1 × PBS buffer was dripped on the POWER and typical superhydrophilic/superhydrophobic substrate.

For the detection of RABV: A 10 μL reaction droplet containing 100 nM of

RABV and 400 nM of H2, H3, and H4 (Detailed sequences see Table S5, Supporting Information) in 10 mM Tris-HCl buffer (PH 7.4) was dripped on the POWER and typical superhydrophilic/superhydrophobic substrate.

6 The molecule diffusion from the surface to the organohydrogel bulk

Rhodamine droplet was dripped on the POWER and hydrogel with a hydrophobic layer for 3 h. The diffusion process was recorded through a laser scanning confocal microscope (Olympus, FV1000-IX81).

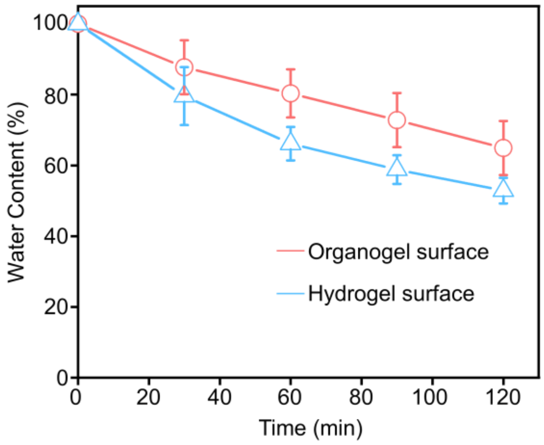

Figure S1. Comparison of the water-retaining performance of organohydrogel with organogel surface and organohydrogel with hydrogel surface at 25 °C and 50% humidity within 120 min. Data represent the mean ± s.d. (N = 3).

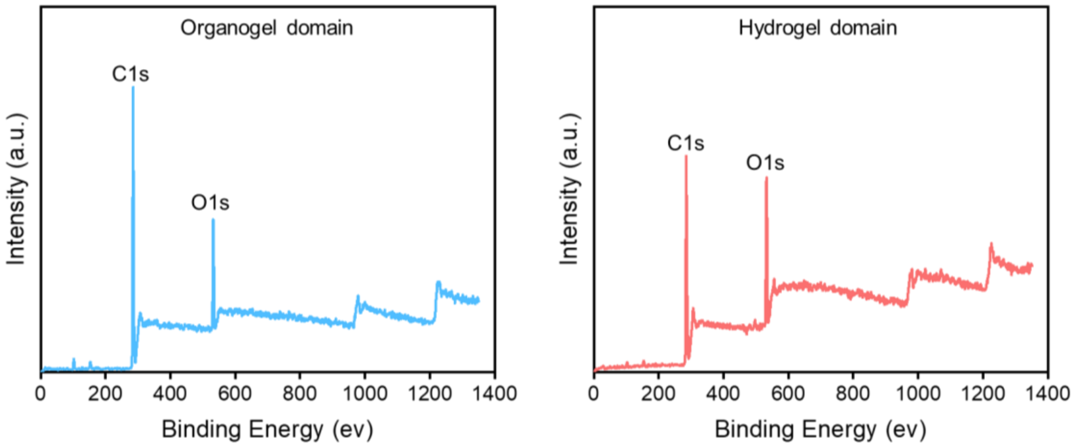

Figure S2. XPS characterization of the organogel and hydrogel domains of the POWER.

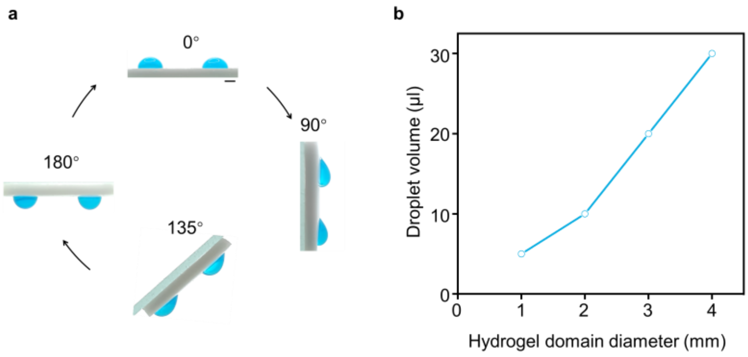

Figure S3. The ability of the POWER to anchor the droplet array. a) Capture of blue-dyed droplet array with different rotation angles (0°, 90°, 135° and 180°). Scale bar, 1 mm. b) Maximum volume of droplets on hydrogel domain with different diameters.

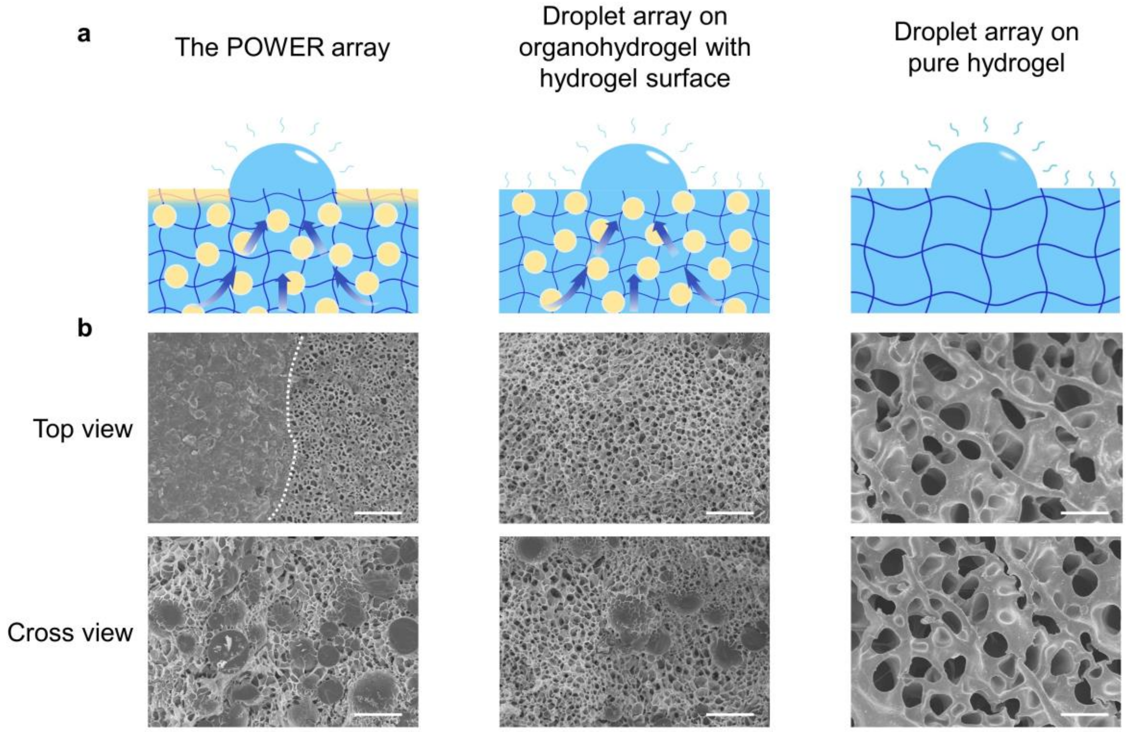

Figure S4. Structure of the POWER array, droplet array on organohydrogel with hydrogel surface and droplet array on pure hydrogel. a) Schematic illustration of the POWER array, droplet array on organohydrogel with hydrogel surface and droplet array on pure hydrogel. b) Corresponding top and cross-view SEM images. Scale bars, 25 μm.

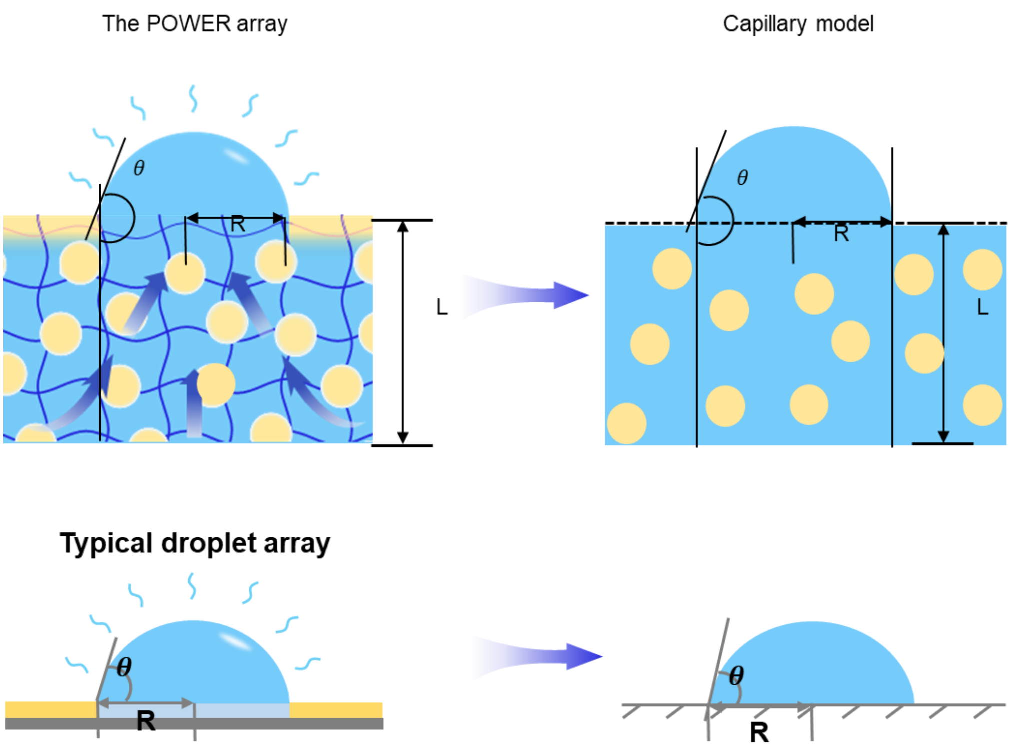

Figure S5. Schematic illustration of the different models of the POWER array and typical superwettable droplet array. A simplified capillary model in porous media was employed for the POWER array to explain the water supply from the bulk. Yellow dots represent the organogel particles. The evaporation process of typical superwettable droplet array conformed to the evaporation model with a forced pinned contact line without water replenishment.

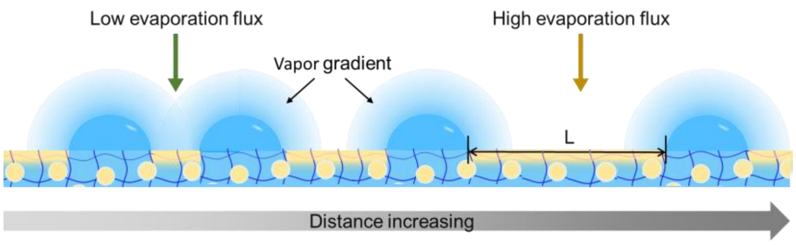

Figure S6. Schematic showing the vapor gradient (blue shades) for two droplets at distance L. The vapor concentration between closer droplets is high, resulting in less evaporation.

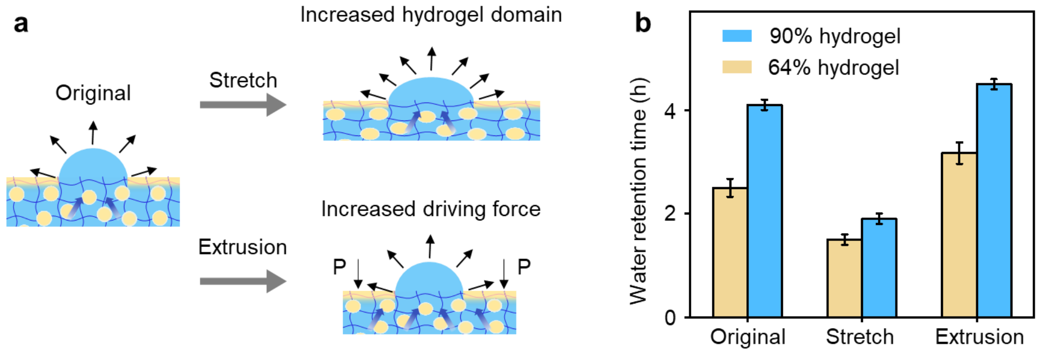

Figure S7. Influence of deformation on the water retention property of the POWER platform. a) Schematic of the original POWER and deformed POWER. Stretching increased the hydrogel domain and squeezing increased the driving force for water supply. b) Comparison of the water retention time of the original and deformed POWER.

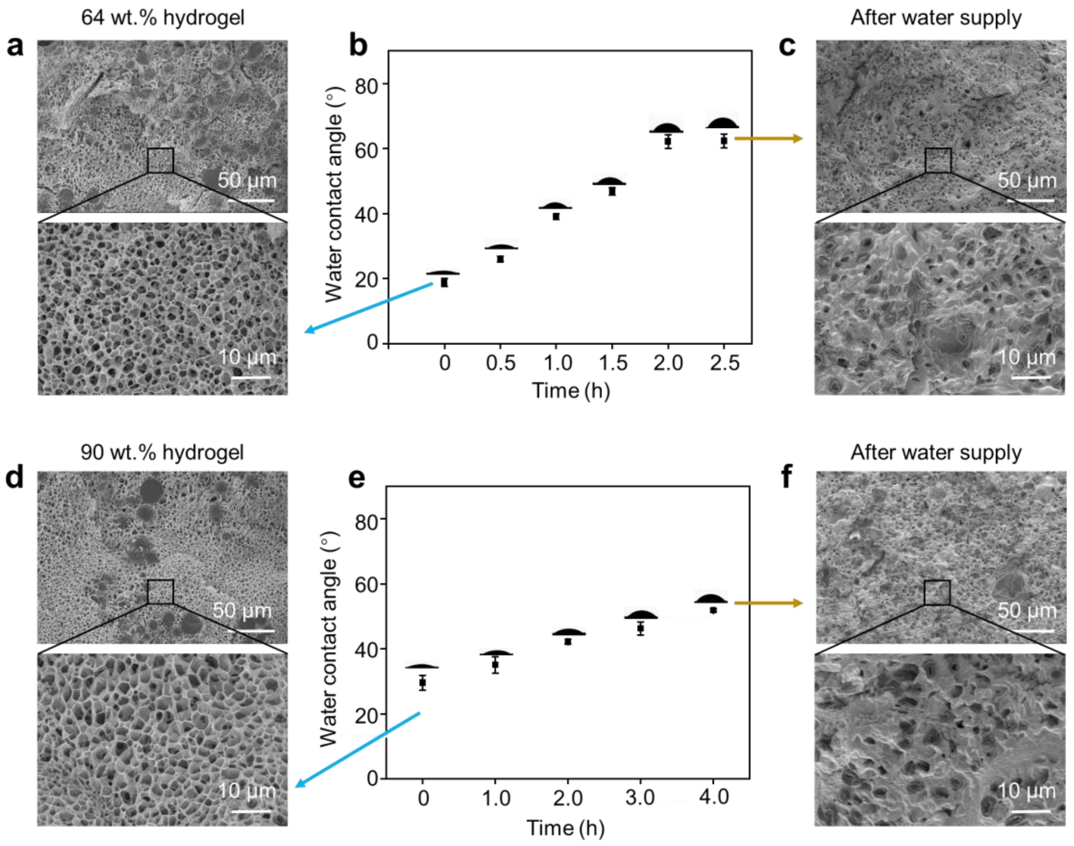

Figure S8. The internal structure and wettability of the POWER platform at different stages of water supply. (a, d) Cross-sectional SEM images showing the structure of the POWER with hydrogel content of 64 wt% and 90 wt%, respectively, before water supply. (b, e) Water contact angle of the POWER bulk at different periods of water supply. (c, f) Cross-sectional SEM images showing the structure of the POWER with hydrogel content of 64 wt% and 90 wt%, respectively, after water supply.

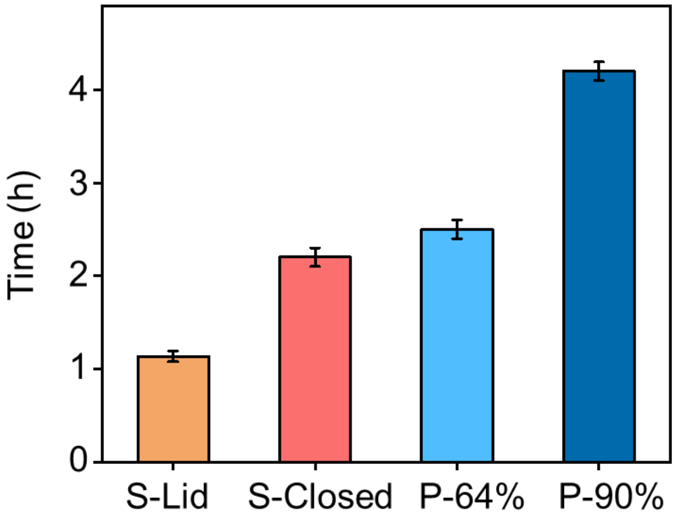

Figure S9. Comparison of the water retention time among traditional superwettable chips covered by a lid (S-Lid) or placed in an enclosed space (S-Closed) and the POWER platform with hydrogel content of 64 wt% (P-64%) or 90 wt% (P-90%).

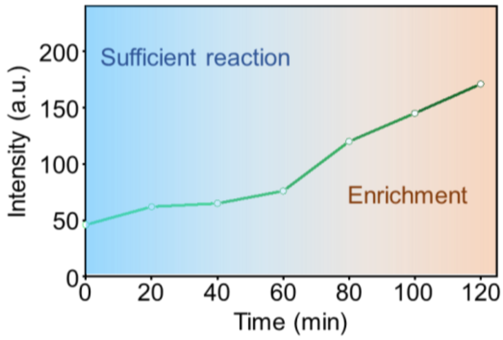

Figure S10. Fluorescence intensity variations of R6G droplets on the POWER were recorded during 120 min at 25 °C and 50% humidity. The excitation wavelength is 492 nm.

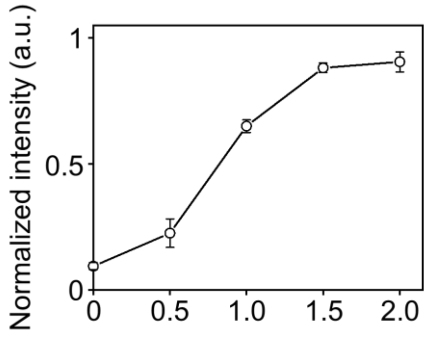

Figure S11. The real-time fluorescence intensity after adding 1 nM target miRNA-21 in the tube.

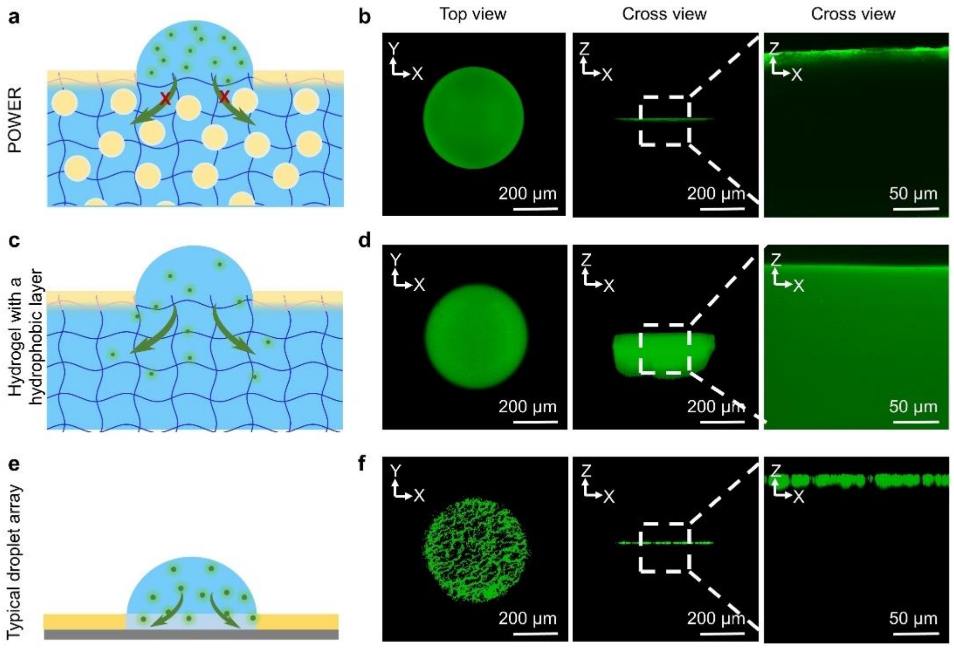

Figure S12. Comparison of molecular diffusion in POWER, hydrogel with a hydrophobic layer and traditional superwettable chips. a) Schematic and b) CLSM images showing the limited diffusion of rhodamine inside the POWER. c) Schematic and d) CLSM images showing the diffusion of rhodamine inside the hydrogel with a hydrophobic layer. e) Schematic and f) CLSM images showing the diffusion of rhodamine inside the traditional superwettable chips composed of inorganic SiO_2.

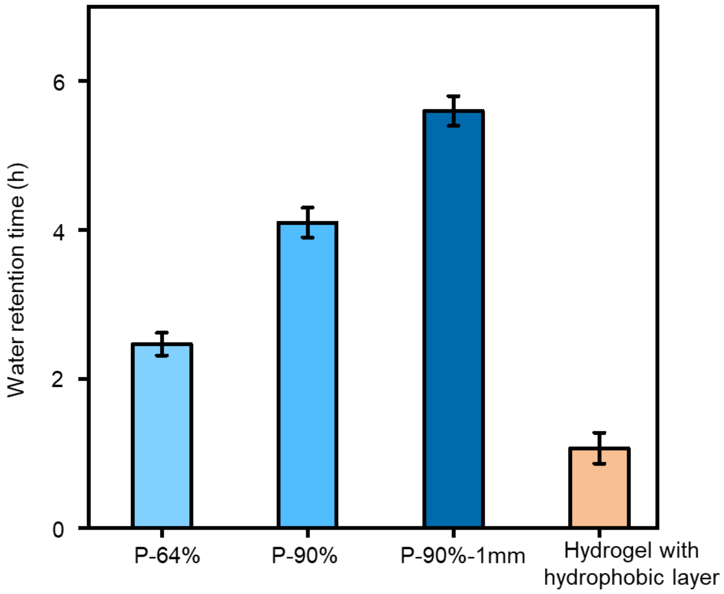

Figure S13. Comparison of the water retention ability of POWER and hydrogel with a surface hydrophobic layer. P-64% and P-90% represent the POWER platform with 64 wt% and 90 wt% hydrogel content and a hydrogel-domain spacing of 15 mm, respectively. P-90%-1mm represents the POWER platform with 90 wt% hydrogel content and a hydrogel-domain spacing of 1 mm.

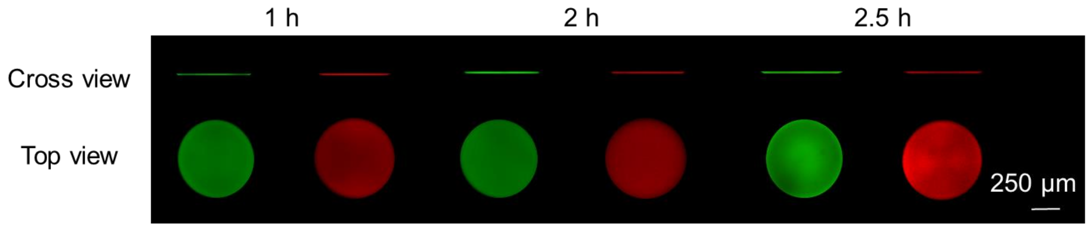

Figure S14. CLSM images showing two individual droplets stained with different fluorescent dyes on adjacent hydrogel domains (500 μm in spacing) and the diffusion of fluorescent molecules in the POWER at different stages. The excitation wavelengths are 492 nm and 450 nm, respectively.

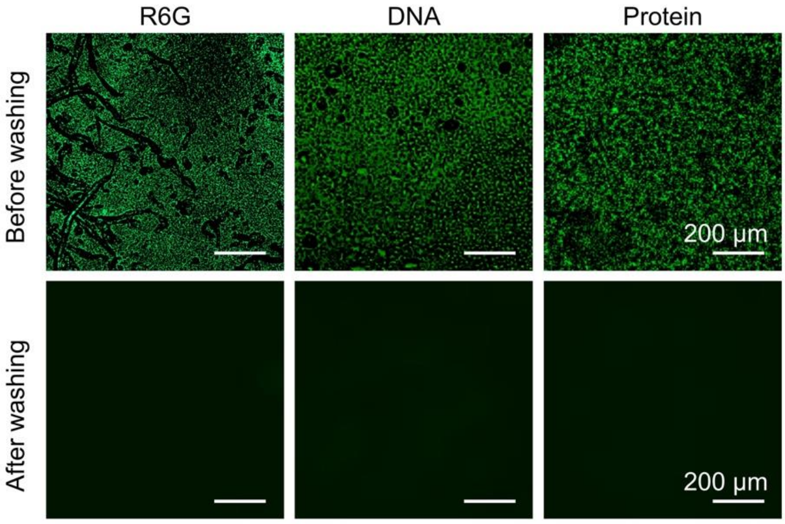

Figure S15. Fluorescence images of the hydrogel domain before and after ultrasonic washing.

Theoretical and experimental analysis of water supply velocity.

The capillary model in porous media (capillary radius of R, CA of θ, porous media length of L) was employed to illustrate the underlying mechanism of water supply from the nether porous organohydrogel (hydrogel domain radius of R, CA of θ, organohydrogel thickness of L). And the evaporation process of typical droplet array can be considered as evaporation with a forced pinned contact line[4].

The theoretical water supply velocity ($Q_{Theo.}$) of the POWER can be calculated by:

$$Q_{Theo.} = -\frac{k}{\mu}\frac{2\pi R\gamma\cos\theta}{L} \tag{S3}$$

Where k is the hydraulic permeability, μ is the dynamic viscosity of the liquid phase, L is the porous media length, γ is the surface tension of the liquid, R is the radius of the capillary, and θ is WCA between the water and the inner wall of the capillary.

The experimental water supply velocity ($Q_{Exp.}$) of the POWER can be calculated by:

$$Q_{Exp.} = M'_{Typical} - M'_{POWER} \tag{S4}$$

Where $M'_{Typical}$ is the mass change rate of the typical droplet array, M'_{POWER} is the mass change per unit time of the POWER array under the same experimental conditions.

Table S1. Comparison with other open droplet arrays for biosensing.

Type	Substrate	Application	Analytes	Time	Sample volume
Oxide	SiO_2/TiO_2	Colorimetric biosensors	PH, Cl^-, Ca^2	5 min[5]	2 μL
		Fluorescence biosensors	R6G	7.5 min[6]	2 μL
Inorganic	Silicon wafer	Surface-enhanced laser-induced breakdown spectroscopy (LIBS)	KCl	22 min[7]	15 μL
Organic	PHEMA-PEDMA	Electrochemical Biosensors	$F_c(M_eOH)_2$	20 s[8]	20 nL
	Cotton/ wax	Colorimetric biosensors	DNA	15 min[9]	20 μL
Mental	ITO/Ti/Au	Electrochemical biosensors	miRNA	30 min[10]	5 μL
	Cu/Ag	SERS biosensors	H_2O_2	10 min[11]	2 μL
This work	PHEA-PLMA	Fluorescence biosensors	miRNA-21<br>RABV	120 min<br>120 min	10 μL

			PDGF-BB	80 min	
		Colorimetric biosensors	VC	20 min	
			VB_1	12 min	
			Fe^{3+}	4 min	

Table S2. The sequences used in the detection of miRNA-21.

Probe	Sequence (5'-3')
HPA	CTCAGCACGGCTCTTTCAACATCAGTCTGATAAGCT TCCAAGGAGCCGTGCTGAGTAT
HPB	TTTCGGCTGGT/FAM/TTATTTTTATTTTATATACTCA GCTT/BHQ1/TCCAGCCGG
HPC	GGAGCCGTTTATTTCTTAGTTTCTCCGGCTGTTTCGG CTCC
Target miRNA-21	UAGCUUAUCAGACUGAUGUUGA
miRNA-141	UAACACUGUCUGGUAAAGAUGG

Table S3. Comparison of POWER-based and in-tubes platforms for miRNA-21 detection through similar SDA systems.

LOD	Volume	Time	Method	Ref.
129.4 pM	200 µL	4.5 h	Fluorescence	12
0.9 nM	200 µL	5 h	Fluorescence	13
1.4 pM	200 µL	2 h	Fluorescence	14
0.1 nM	200 µL	2 h	Fluorescence	15
10 pM	200 µL	4 h	Fluorescence	16
0.4 pM	10 µL	2 h	Fluorescence	This work

Table S4. The sequences used in the detection of Platelet-derived growth factor-BB (PDGF-BB).

Probe	Sequence (5’-3’)
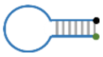 H_1	FAM/CGTTCACAGGCACAGGCTACGGCACGTAGAGC ATCACCATGATCCTGTGAACG/BHQ1
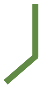 DNA_1	CGTTCACAGGATCATG
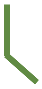 DNA_2	CATGATCCTGTGCCTGTGAA

Table S5. The sequences used in the detection of rabies virus (RABV).

Probe	Sequence (5’-3’)
H_2	FAM/ACATAAGTTTCAGTTATTAGTCCATCCAATCAC AACTGGACTAATAACTGAAAC/BHQ1
H_3	GTTATTAGTCCAGTTGTGATTGGATGAAACTTATGT TCCAATCACAACTGGACT
H_4	GTTGTGATTGGAACATAAGTTTCATGGACTAATAAC TGAAACTTATGTTCCAAT
RABV	TGGACTAATAACTGAAACTTATGT

Reference

1. R. Xi, Y. Wang, X. Li, X. Zhang, X. Du, *J. Colloid Interface Sci.* **2020**, *563*, 42-53.

2. H. Peng, X. F. Li, H. Zhang, X. C. Le, *Nat. Commun.* **2017**, *8*, 14378.

3. H. Dacres, M.M. Dumancic, I. Horne, S. C. Trowell, *Biosens. Bioelectron.* **2009**, *24*, 1164-1170.

4. K. Gleason, S. A. Putnam, *Langmuir* **2014**, *30*, 10548-10555.

5. X. He, T. Xu, Z. Gu, W. Gao, L-P. Xu, T. Pan, X. Zhang, *Anal. Chem.* **2019**, *91*, 4296-4300.

6. J. Hou, H. Zhang, Q. Yang, M. Li, Y. Song, L. Jiang, 2014, *Angew. Chem. Int. Ed.* *53*, 5791-5795.

7. D. Bae, S.-H. Nam., S.-H. Han, J. Yoo, Y. Lee, *Spectrochimica. Acta B.* 2015, *113*, 70-78.

8. H. Zhang, T. Oellers, W. Feng, T. Abdulazim, E. N. Saw, A. Ludwig, P. A. Levkin, N. Plumere, *Anal. Chem.* **2017**, *89*, 5832-5839.

9. Wu, T. Xu, L.-P. Xu, Y. Huang, W. Shi, Y. Wen, X. Zhang, *Biosens. Bioelectron.***2016**, *86*, 951-957.

10. T. Xu, Y. Song, W. Gao, T. Wu, L.-P. Xu, X. Zhang, S. Wang, *ACS Sens.* **2018**, *3*, 72-78.

11. Z. Yu, Y. Park, L. Chen, B. Zhao, Y. M. Jung, Q. Cong, *ACS Appl. Mater. Interfaces* **2015**, *7*, 23472-23480.

12. G. Jie, Y. Zhao, X. Wang, C. Ding, *Sensor. Actuat. B-Chem.* **2017**, *252*, 1026-

1034.

13. Y. Wan, G. Li, L. Zou, H. Wang, Q. Wang, K. Tan, X. Liu, F. Wang, *Anal. Chem.* **2021**, *93*, 11052-11059.

14. R. Liu, S. Zhang, T. T. Zheng, Y. R. Chen, J. T. Wu, Z. S. Wu, *ACS Nano* **2020**, *14*, 9572-9584.

15. C. Li, M. Luo, J. Wang, H. Niu, Z. Shen, Z. S. Wu, *ACS Sens.* **2020**, 5, 2378-2387.

16. C. Xue, S. X. Zhang, C. H. Ouyang, D. Chang, B. J. Salena, Y. Li, Z. S. Wu, *Angew. Chem. Int. Ed.* **2018**, *57,* 9739-9743.
